# 1302. Impact of Insurance Coverage on the Route of Antibiotic Treatment for Forefoot Osteomyelitis

**DOI:** 10.1093/ofid/ofad500.1141

**Published:** 2023-11-27

**Authors:** Austin Ritter, Anais Ovalle, Zainah Hadeel

**Affiliations:** Brown University/Kent Hospital, Baltimore, Maryland; Care New England/Brown University, West Lebanon, New Hampshire; Kent-County Memorial Hospital, Warwick, Rhode Island

## Abstract

**Background:**

Recent studies suggest oral antibiotics can be a non-inferior alternative to intravenous antibiotics for treating bone and joint infections. In this study, we aimed to investigate whether insurance type affects the choice of antibiotic treatment routes and whether the route of antibiotic treatment influences the delivery of home health services.

**Methods:**

We conducted a retrospective chart review of non-bacteremic patients who underwent amputations for forefoot osteomyelitis with residual signs of infection on proximal bone cultures and were discharged home from the hospital. Of 461 patients scheduled for hospital follow-up visits for bone and joint infections in our community practice infectious diseases clinic from January 2019 to December 2022, 40 met the inclusion criteria. Results were analyzed with multivariable logistic regression, selecting models with the lowest Akaike Information Criterion.

**Figure:**
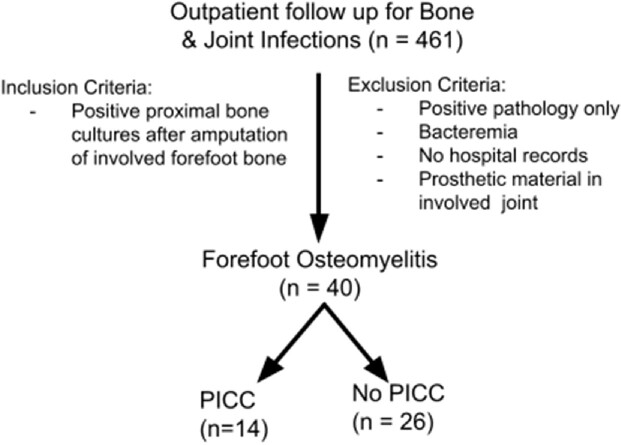

**Results:**

The placement of PICC lines decreased significantly over time (OR 0.34, 95% CI: 0.14 - 0.71, p = 0.01), mirroring national trends favoring increased oral antibiotic use. After controlling for the year of treatment, we observed a trend towards patients with Medicaid insurance more frequently receiving IV antibiotics (OR 4.8, 95% CI: 1.0 - 29, p = 0.06). We found no evidence that the antibiotic administration route led to differences in the delivery of home health services at the time of discharge (p = 0.99).

PICC Placement Percentage by Year and Insurance

**Figure:**
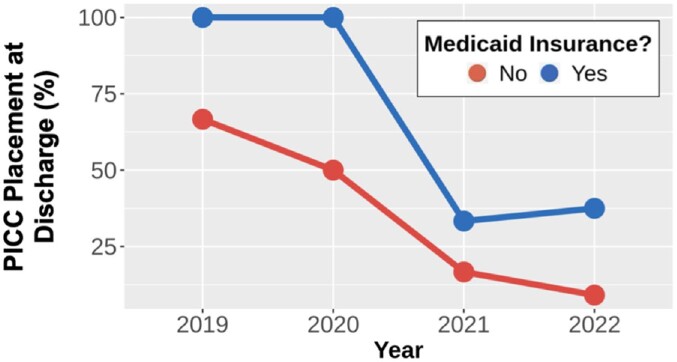

Percentage of PICC line Placement by year in patients with and without Medicaid Insurance during hospital encounter

PICC Placement Frequency by Insurance and Home Nursing

**Figure:**
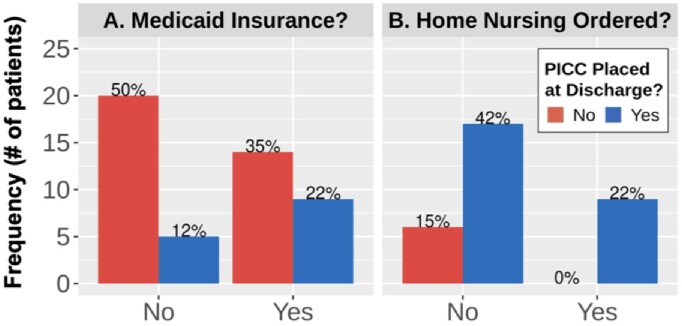

Frequency of PICC placement in patients during hospital encounters based on (A) if a patient has Medicaid insurance and (B) if home nursing is ordered at discharge

Organisms Cultured by Year

**Figure:**
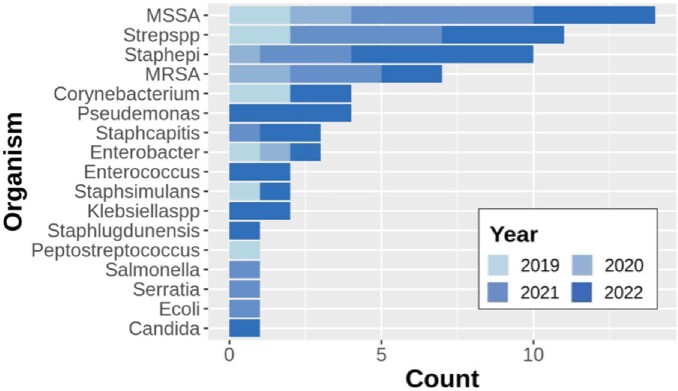

Frequency of organisms cultured on proximal bone cultures by year of study

**Conclusion:**

Our findings suggest a trend towards patients with Medicaid insurance more frequently receiving IV antibiotics, despite the higher direct and indirect costs. It remains unclear whether this trend is due to differences in the severity of infections or microbiological resistance patterns. Future studies should assess the interaction of psychosocial and microbiological factors in influencing shared decisions regarding the route of antibiotic treatment outside of clinical trial settings.

Organisms Cultured by Antibiotic Route, Insurance Type, and Home Nursing Orders

**Figure:**
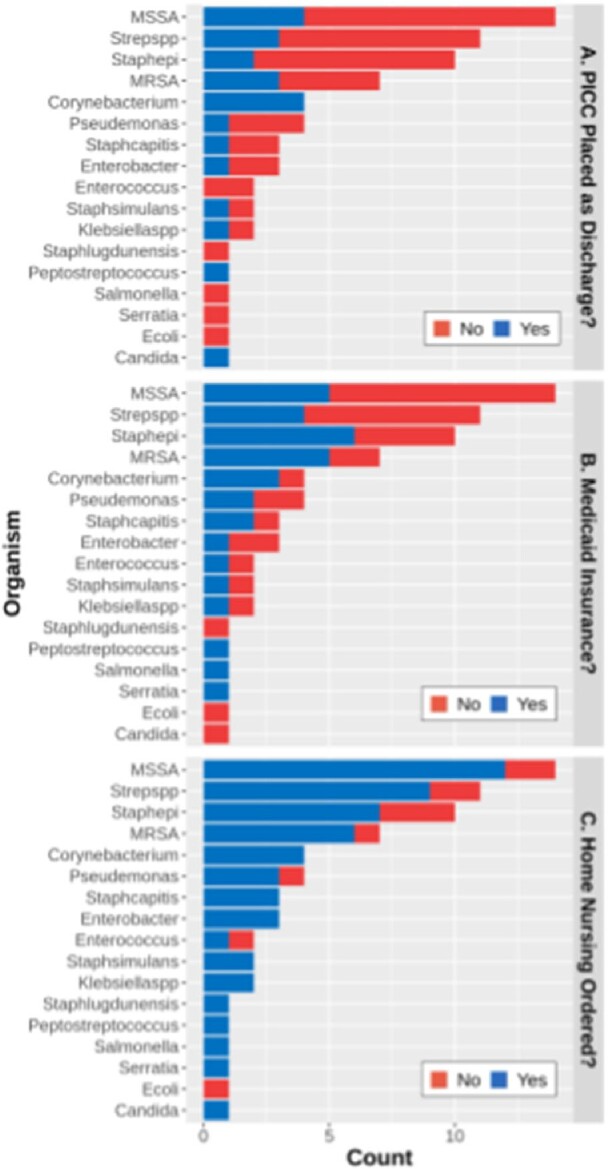

Frequency of PICC line placement (A), Insurance coverage (B), and ordered for home nursing at discharge (C) by organisms cultured from proximal bone margins

**Disclosures:**

**All Authors**: No reported disclosures

